# Thymoma resection and myasthenia gravis: what is the neurological outcome in patients older than 65 years?

**DOI:** 10.1007/s13304-024-01937-w

**Published:** 2024-07-09

**Authors:** Filippo Lococo, Carolina Sassorossi, Giulio Maurizi, Gloria Santoro, Raffaele Iorio, Silvia Falso, Elisa Meacci, Antonio Giulio Napolitano, Maria Teresa Congedo, Giacomo Cusumano, Beatrice Trabalza Marinucci, Giacomo Argento, Marco Chiappetta, Erino Angelo Rendina, Stefano Margaritora

**Affiliations:** 1 Thoracic Surgery Unit, Università Cattolica del Sacro Cuore, Largo F.Vito 1, Rome, Italy; 2 Thoracic Surgery Unit, Fondazione Policlinico Universitario A. Gemelli IRCCS, Rome, Italy; 3 Department of Medical-Surgical Science and Translational Medicine, Sapienza University of Rome, Rome, Italy; 4 Division of Thoracic Surgery, Sant’Andrea University Hospital, Rome, Italy; 5 UOC di Chirurgia Generale, Fondazione Policlinico Universitario A. Gemelli IRCCS, 00168 Rome, Italy; 6 Neurology Unit, Fondazione Policlinico Universitario A. Gemelli IRCCS, Rome, Italy; 7 Department of Neuroscience, Università Cattolica del Sacro Cuore, Rome, Italy; 8 Thoracic Surgery Unit, Policlinico-San Marco Hospital, University of Catania, Catania, Italy

**Keywords:** Myasthenia gravis therapy, Surgery, Thymectomy

## Abstract

To increase the neurological results in patients older than 65 years with myasthenia gravis after thymectomy, we retrospectively analysed this outcome in a large bicentric cohort of patients with myasthenia gravis (MG)years, for which surgery was indicated for a concurrent thymoma. From 1/2000 to 2/2022, 502 patients underwent thymectomy for thymic epithelial tumours (TETs) in two high-volume Institutions (167aged more than 65 years). Among them, 66 patients were affected by TET and MG, representing our final study group. The mean age for MG onset was 68.3 ± 6 years.At surgery, the Osserman score 2 was the most diffuse in our cohort (43, 65.1%), followed by 1 (20, 30.3%). In 11 cases, the MG diagnosis coincided with thymoma diagnosis. In the other cases, the interval between MG diagnosis and surgery was 1.7 years ± 1.9. The most common surgical approach was sternotomy (41,62.1%), followed by RATS (14,21.2%). The most frequent TNM stage was T1N0 (75.7%) and most patients had WHO type-B tumour. After radical thymectomy, 58 patients (88%) reported a significant neurological improvement. According to MGFA-PIS, after surgery we had 4 (6%) complete stable remission, 11 (16.7%) pharmacological remission, 43 (65.2%) minimal manifestation, 2 (3%) worsening/death for MG, and 5 (7.6%) unchanged. No association was found between neurological outcome and age of MG onset, kind of pharmacological therapy before surgery, surgical approach (sternotomy vs others), tumour dimension, the ITMIG stage and the preoperative Osserman score. For MG and thymoma-afftected patients over 65 years, thymectomy seems to be an effective treatment to improve neurological symptoms. We suggest to set up clinical trials to explore the neurological efficacy of mini-invasive thymectomy in clinically selected MG patients aged over 65 years.

## Introduction

Myasthenia gravis (MG) is a chronic neuromuscular disorder caused by autoantibodies against neuromuscular junction proteins [1, 2].

Presently, there are no well-defined guidelines for defining the treatment strategies. The indication for the therapies are collected in a consensus guidance, which has been updated in 2020. In particular, concerning the surgical option, the International Consensus Guidance for Management of Myasthenia Gravis suggests thymectomy as a treatment for nonthymomatous, generalized MG patients with AChR-Ab, younger than 65, to improve clinical outcomes and to minimize immunotherapy requirements [3]. This recommendation came out from a multicenter, randomized, rater-blinded trial of thymectomy in MG patients younger than 65 years with AChR-positive (AChR-Ab +) generalized nonthymomatous MG of < 5 years duration [3]. In this cohort, the thymectomy was performed via sternotomy and resulted to be useful in the reduction of glucocorticoid therapy and immunosuppressive therapy such as azathioprine. As a consequence of trial results, surgery for MG is not recommended in patients aged more than 65 years. However, in the recent literature some authors are advocating the need of extending the indication of surgery to older patients, especially after the consolidation of the mini-invasive technique (VATS, RATS) to perform a radical thymectomy [4].

To increase the neurological results in patients older than 65 years with myasthenia gravis after thymectomy, we retrospectively analysed this outcome in a large bicentric cohort of patients with MG aged more than 65 years. In particular, in our cohort, surgery had an oncological indication, and patients were both affected by thymic epithelial tumour and MG. For few cases, in which patients presented with higher Osserman score (i.e. 3 or 4), indication to surgery was with the purpose of a possible neurological benefit.

The primary end point was the neurological outcome, analysed as the MGFA-PIS. Furthermore, we also evaluated factors associated with the improvement of the neurological outcome.

## Methods

From 1/2000 to 2/2022, a total of 502 patients underwent thymectomy for thymic epithelial tumours in two high-volume Institutions (Policlinico A. Gemelli and Azienda Ospedaliera Sant’Andrea). Among them, 167 were aged more than 65 years and finally 66 patients were affected with thymoma and MG, representing our final study group.

Preoperative oncological assessment included contrasted CT total body scan and magnetic resonance, if indicated in case of suspected infiltration of neighbouring organs. PET/CT was not performed in all cases, according to the study period and surgeon’s choice. Thymic epithelial tumour was suspected on the base of radiological and radiometabolic characteristics, and confirmatory biopsy was performed only in case of doubt before surgery. MG diagnosis included the anti-acetylcholine receptor, anti-MuSK antibodies and electromyography (EMG) and neurological clinical evaluation made by Osserman score. All patients were positive for anti-AChR Abs. Titres were measured at MG diagnosis and not routinely evaluated during follow-up, and there is still lack of evidence that titration is associated with disease severity.

The management of neurological therapy for surgery and the definition of the more proper “therapeutic timing” in which surgery could be performed without elevated risk for the patients were discussed with the neurologists and anaesthesiologists. Preoperative neurological therapy was tailored according to patients’ clinical condition. Based on current guidelines [3], the treatment regimen included cholinesterase inhibitors as first-line medication and prednisone was started at 0.5–1 mg/kg daily dosage in patients unresponsive to pyridostigmine. No change in steroid therapy was made before surgery. Surgery is scheduled only when neurological symptoms are controlled and steroid therapy is maintained until new evaluation after surgery. Analysis of the respiratory function was also performed before surgery by standard spirometry. Before surgery, all cases were discussed in a multidisciplinary meeting, composed of surgeons, oncologists, neurologists and radiotherapists.

All the patients were operated under general anaesthesia. Surgery was performed by median sternotomy or thoracotomy. After the introduction of minimally invasive techniques, also video-assisted thoracic surgery (VATS) and robotic resection were performed in selected cases, mainly for tumours smaller than 3 cm and for thymomas appearing not invading the surrounding structures. All the operations were performed with the oncological aim of radical resection (radical thymectomy, including thymus and perithymic fat). In case of suspected infiltrations, where possible, infiltrated structures were removed en bloc with the tumour and the area of removal marked with titanium clips.

Masaoka–Koga stage classification was adopted for pathological assessment and the study group was divided into low-risk thymomas (types A, AB, and B1) and high-risk thymomas (types B2, B3, and thymic carcinomas) [A].

The MGFA post- intervention status (PIS) was analysed to evaluate the effect of surgery in these patients [B] Due to the rarity of the disease and to the unbalanced categories, we grouped the classes as summarized herein: good neurological response (complete stable remission + pharmacological remission), minimal manifestation (all the degrees of minimal manifestation), and no response (unchanged + worsening/death due to MG). Neurological examination was performed during the postoperative period, at 1 month after surgery and the next visits were scheduled according to the patient’s condition.

### Statistical analysis

Explanatory descriptive analysis (EDA), crucial to understand the structure and characteristics of the data, was performed to better understand variable distributions and frequencies through a sample of 66 patients from multicentric hospitals. A preliminary analysis wais conducted to calculate frequencies, means, medians, standard deviations, and interquartile ranges, which provided a solid foundation for further analysis. Correlations among variables were considered to overview covariance (polycor and corrplot library in R).

Nevertheless, based on the absence of statistical significance and strong correlations between every single feature vs MGFA-PIS (outcome), we performed a supervised machine learning method of classification with the Random Forest algorithm. Random forest is an excellent choice for handling categorical variables. This algorithm is known for its flexibility and ability to handle both classification and regression problems. It combines the results of multiple decision trees to obtain a more accurate estimate.

First of all, we considered dichotomous neurological response conditions: a good response (15 patients) that was composed of CSR and PR and the minimal manifestation response (43 patients), composed of all the degrees of MM. Therefore, the data of a total of 58 patients were analysed.

To balance the sample, we uploaded functions to deal with binary classification problems, working with ROSE (Random Over-Sampling Examples) package implemented in R (https://cran.r-project.org) and finally generated synthetic balanced samples. This package is a bootstrap-based technique which aids inthe task of binary classification in the presence of rare classes [6, 7]. Handling class imbalance using the ROSE algorithm, it is possible to address the issue of class imbalance in the dataset. This helps ensure a more balanced representation of classes during model training.

The balanced sample was divided into 15 patients for good neurological response vs 43 for minimal manifestation. Starting from this dataset, we performed a classification analysis and designed a random forest (randomForest, caret, rpart, fancyrpart package in R) dividing the sample into 70% in the training set and the remaining 30% as a test set. Furthermore, leave-one-out cross-validation (LOOCV) estimate of the area under the curve (AUC) of a classifier trained on balanced data provided 70% accuracy and 30% precision.

Finally, we considered survival analysis for the total sample and analysed the Kaplan–Meier method with a log-rank test (libraries MASS, survival, survminer, and ggplot2 in R).

## Results

The main demographic, clinical, surgical, and neurological features are described in Table 1. In particular, the mean age for MG onset was 68.3 ± 6. The mean interval between MG onset and surgery was 173 ± 1 years. Anti-AchR Ab was found in 52 (78.8%) patients, and none of them presented with anti-MuSK Ab. Mean age at surgery was 70.1 ± 5.5 years. Preoperative Osserman score 2 was the most diffuse in our cohort (35 (53%) 2a, and 7 (10.6%) 2b), followed by Osserman score 1 (20 patients, 30.3%). In eght cases, the Osserman score was higher (i.e. 3 or 4) and in these cases, together with the oncological indication, surgery was done also with the purpose of a neurological benefit.

**Table 1:** Main clinical and pathological characteristics (*N* = 66)

Age at surgery—years	
Mean; SD	70.1 ± 5.5
Sex—*n* (%)	
M	23 (34.8%)
F	43 (65.1%)
Myasthenia gravis (MG)—*n* (%)	
YES	66 (100%)
NO	0 (0%)
Ab anti-Ach receptor—*n* (%)	
YES	52 (78.8%)
NO	8 (12.2%)
NA	6 (9.0%)
Ab anti-MuSK—*n* (%)	
YES	0 (0%)
NO	66 (100.0%)
Age at MG onset—*n* (%)	
Mean ± SD	68.3 ± 6
Type of medication—*n* (%)	
Corticosteroid	21 (31.8%)
Corticosteroid–pyridostigmine	10 (15.1%)
Corticosteroid–pyridostigmine-Immunosoppressor (azathioprine, mycophenolate mofetil, cyclosporine or cyclophosphamide)	20 (30.3%)
Immunosoppressor (azathioprine, mycophenolate mofetil, cyclosporine or cyclophosphamide)	2 (3.1%)
Pyridostigmine	13 (19.7%)
Type of surgery—*n* (%)	
STERNOTOMY	41 (62.1%)
RATS	14 (21.2%)
THORACOTOMY	7 (10.6%)
VATS	4 (6.1%)
Pericardial resection—*n* (%)	
YES	10 (15.2%)
NO	56 (85.8%)
Pleural resection—*n* (%)	
YES	15 (22.7%)
NO	51 (77.3%)
Lung resection—*n* (%)	
YES	10 (15.2%)
NO	56 (84.8%)
Tumour dimension (cm)	
Mean ± SD	4.7 ± 3.3
Capsule microscopic infiltration—*n* (%)	
NO (0)	21 (31.8%)
Only CAPSULE (1)	16 (24.3%)
CAPSULE and FAT (2)	29 (43.9%)
Pericardial Infiltration—*n* (%)	
YES	7 (10.6%)
NO	59 (89.4%)
Classification T—*n* (%)	
T1	50 (75.7%)
T2	5 (7.6%)
T3	10 (15.2%)
T4	1 (1.5%)
R status—*n* (%)	
R0	64 (96.9%)
R1	2 (3.1%)
Masaoka classification—*n* (%)	
1	20 (30.3%)
2	36 (54.5%)
3	6 (9.1%)
4	4 (6.1%)
Histology—*n* (%)	
Low risk (A, B1)	35 (53.0%)
High risk (B2, B3)	31 (47.0%)
MGFA	
I	16 (24.2%)
IIA	32 (48.5%)
IIB	8 (12.2%)
IIIA	6 (9.1%)
IIIB	2 (3%)
IV	2 (3%)
V	0
MG Osserman score	
1	16 (24.2%)
2a	35 (53%)
2b	7 (10.6%)
3	6 (9.1%)
4	2 (3.1%)
Adjuvant therapy—*n* (%)	
NO	40 (60.7%)
CT	4 (6.0%)
RT	22 (33.3%)
MGFA-PIS—*n* (%)	
CSR	4 (6.1%)
PR	11(16.7%)
MM	43(65.2%)
U	5 (7.5%)
W/D	2 (3%)
NA	1 (1.5%)
Neurological outcome—simplified MGFS-PIS classification (%)	
Good response (CSR + PR)	15 (22.8%)
Minimal manifestation response (MM)	43 (65.2%)
U/W/D	7 (10.5%)
NA	1 (1.5%)

The most frequent kind of medical treatment of MG was steroid alone (31.8%) or in combination with immunosuppressor and Pyridostigmine (30.3%).

In 11 cases, the MG diagnosis coincided with thymoma diagnosis, while in the other cases, the interval between MG diagnosis and surgery was 1.7 years ± 1.9. The most common surgical approach was sternotomy (#41, 62.1%), followed by RATS (#14, 21.2%) as reported in Table 1. After surgery, we had 64 (97%) R0 resections. The most frequent TNM stage was T1N0 (75.7%). Adjuvant therapy was necessary in 36 (54.5%) patients. In all the cases in which radiotherapy was administered, when in doubt of a microscopic residual disease, during the postoperative multidisciplinary meeting, the decision to perform radiotherapy on the bed of the tumour was made in consultation with the radiotherapist and oncologist,

According to MGFA-PIS, a total of 58 patients (88%) reported a significant neurological improvement after radical thymectomy, epresenting the primary aim of the present analysis.

In detail, after surgery, we observed that 4 patients (6%) had complete stable remission, 11 (16.7%) presented with pharmacological remission, 43 (65.2%) had only minimal manifestations, 2 (3%) had worsening of/death due to MG and finally 5 (7.6%) were unchanged.

In particular, out of 16 patients with preoperative Osserman score 1, 2 obtained a complete stable response, 11 minimal manifestation, and 3 pharmacological remission. Out of 35 patients’ stage 2a before surgery, 2 obtained a CSR, 28 obtained a minimal manifestation response, 3 a partial response, and 2 was unchanged. Out of the seven patients staged 2b before surgery, six obtained pharmacological remission and one was unchanged. The two patients who presented a worsening in their condition or died were one with stage 2a and the other with 2b.

At the univariate analysis, no statistically significant correlation was found among the variable considered and the subgroups for the neurological response (Table 2). In particular, for the univariate analysis, from the whole population of 66 patients, 7 with unchanged or worsened status were excluded, due to the small number compared with the rest of the population. Then the cohort was divided in two MGFA-PIS neurological responses the sub-sample of 58 patients that got a response, divided in two groups; minimal manifestation response vs good response (CSR + PR), as already described in methods section (Table 2).

**Table 2:** Univariate analysis based on good response vs minimal manifestation response

Neurological response	Levels	Good response (Tot. 15)	Minimal manifestation (Tot. 43)	*p* value
Sex	F	7 (46.7%)	31 (72.1%)	0.14
	M	8 (53.3%)	12 (12.9%)	
Age at surgery	Mean (SD)	69.5 (4.9)	71.1 (5.9)	0.41
Ab anti-Ach receptor	NO	1 (8.3%)	6 (15.0%)	0.91
	YES	11 (91.7%)	34 (85.0%)	
Age at MG onset	Mean (SD)	66.8 (4.4)	69.5 (6.5)	0.36
Type of medication for MG	corticosteroid	6 (40.0%)	13 (30.2%)	0.82
	Corticosteroid pyridostigmine intolerant: immunosoppressor	0 (0.0)	1 (2.3%)	
	Corticosteroid pyridostigmine immunosoppressor	4 (26.7%)	11 (25.6%)	
	Corticosteroid pyridostigmine	3 (20.0%)	6 (14.0%)	
	Immunosoppressor	0 (0.0)	2 (4.7)	
	Pyridostigmine	2 (13.3)	10 (23.3%)	
Surgical approach	Rats	3 (20)	8 (18.6%)	0.18
	Sternotomy	12 (80.0%)	24 (55.8%)	
	Thoracotomy	0 (0.0)	7 (16.3%)	
	VATS	0 (0.0)	4 (9.3%)	
Minimally invasive vs open approach	Minimally invasive	3 (20%)	12 (27.9%)	0.7
	Open approach	12 (80%)	31 (72.1%)	
Pericardial resection	NO	13 (86.7%)	36 (85.7%)	1
	YES	2 (13.3%)	6 (14.3%)	
Pleural resection	NO	12 (80.0%)	33 (76.7%)	1
	YES	3 (20.0%)	10 (23.3%)	
Lung resection	NO	14 (93.3%)	35 (81.4%)	0.49
	YES	1 (6.7%)	8 (18.6%)	
TNM	T1N0	12 (80.0%)	32 (74.4%)	0.88
	T2N0	1 (6.7%)	2 (4.7%)	
	T3N0	2 (13.3%)	8 (18.6%)	
	T4N0	0 (0.0)	1 (2.3%)	
Tumour dimension	Mean (SD)	5.9 (3.3)	4.6 (3.3)	0.08
Capsule microscopic infiltration	NO	5 (33.3%)	16 (37.2%)	0.61
	Only capsule	5 (33.3%)	9 (20.9%)	
	Capsule and fat	5 (33.3%)	18 (41.9%)	
Pericardial infiltration	NO	13 (86.7%)	39 (90.7%)	1
	YES	2 (13.3%)	4 (9.3%)	
Pleural infiltration	NO	14 (93.3%)	38 (88.4%)	0.95
	YES	1 (6.7%)	5 (11.6%)	
Lung infiltration	NO	15 (100.0%)	40 (93.0%)	0.71
	YES	0 (0.0)	3 (7.0%)	
Vascular infiltration	NO	14 (93.3)	40 (93.0%)	1
	YES	1 (6.7%)	3 (7.0%)	
Disease residue	R0	14 (93.3%)	42 (97.7%)	1
	R1	1 (6.7%)	1 (2.3%)	
Histology	High risk	8 (53.3%)	18 (41.9%)	0.64
	Low risk	7 (46.7%)	25 (58.1%)	
ITMIG stage	I	2 (40.0%)	11 (55.0%)	0.66
	II	1 (20.0%)	1 (5.0%)	
	IIIA	2 (40.0%)	7 (35.0%)	
	IIIB	0 (0.0)	1 (5.0%)	
MG Osserman score	Score 1	5 (33.3%)	11 (25.6%)	0.82
	Score 2	9 (60.0%)	28 (65.1%)	
	Score 3	1 (6.7%)	4 (9.3%)	
	Score 4	0 (0.0%)	0 (0.0%)	
Adjuvant treatment	NO	6 (40.0%)	22 (51.2%)	0.73
	RT	8 (53.3%)	18 (41.9%)	
	CT	1 (6.7%)	3 (7.0%)	

Interestingly, no association was found between the neurological outcome and the age of MG onset, the kind of pharmacological therapy performed before surgery, the kind of surgical approach (minimally invasive vs open approach), tumour dimension, the ITMIG stage, and the preoperative Osserman score.

We also analysed the interval between the onset of MG and surgery, dividing patients into two groups, the ones that have been operated within 12 months and the ones operated after 12 months from the onset of MG. No statistically significant difference was found between the two groups in terms of neurological outcome (MGFA-PIS) (p value 0.8).

**Fig. 1:**
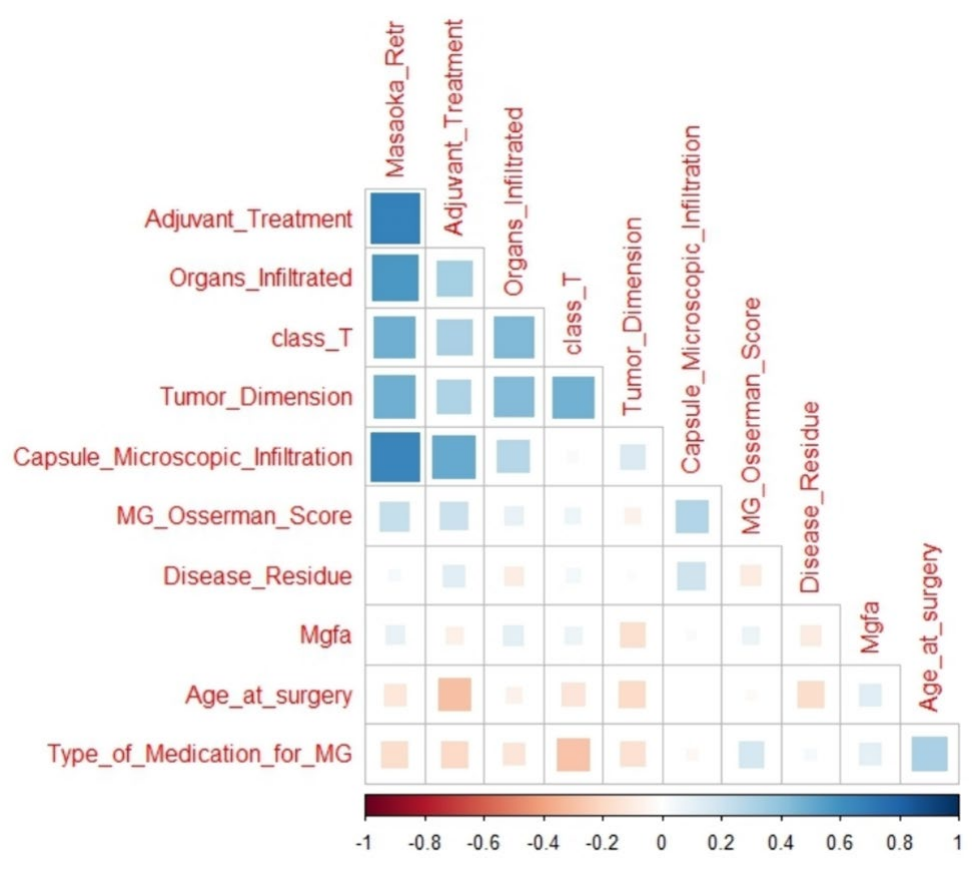
Correlation matrix between clinical–pathological characteristics to identify direct or indirect association between variables

**Fig. 2:**
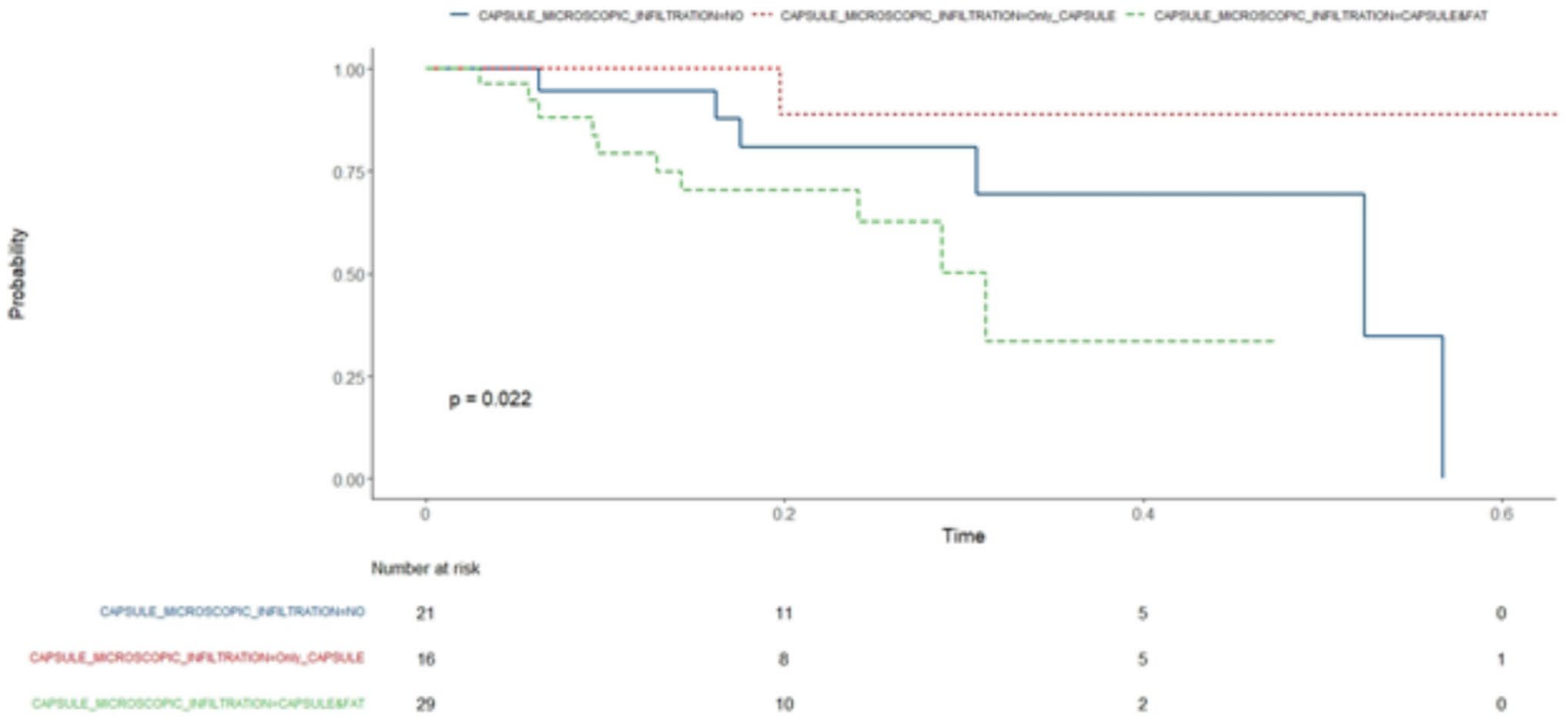
Capsule microscopic infiltration Kaplan–Meier test examining survival trends. The Kaplan–Meier test based on capsule microscopic infiltration offers valuable insights into survival trends among patients with different degrees of capsule infiltration in their tumours. This test categorizes patients into groups based on the presence or absence of microscopic infiltration of the tumour capsule and evaluates their survival probabilities over time

**Fig. 3:**
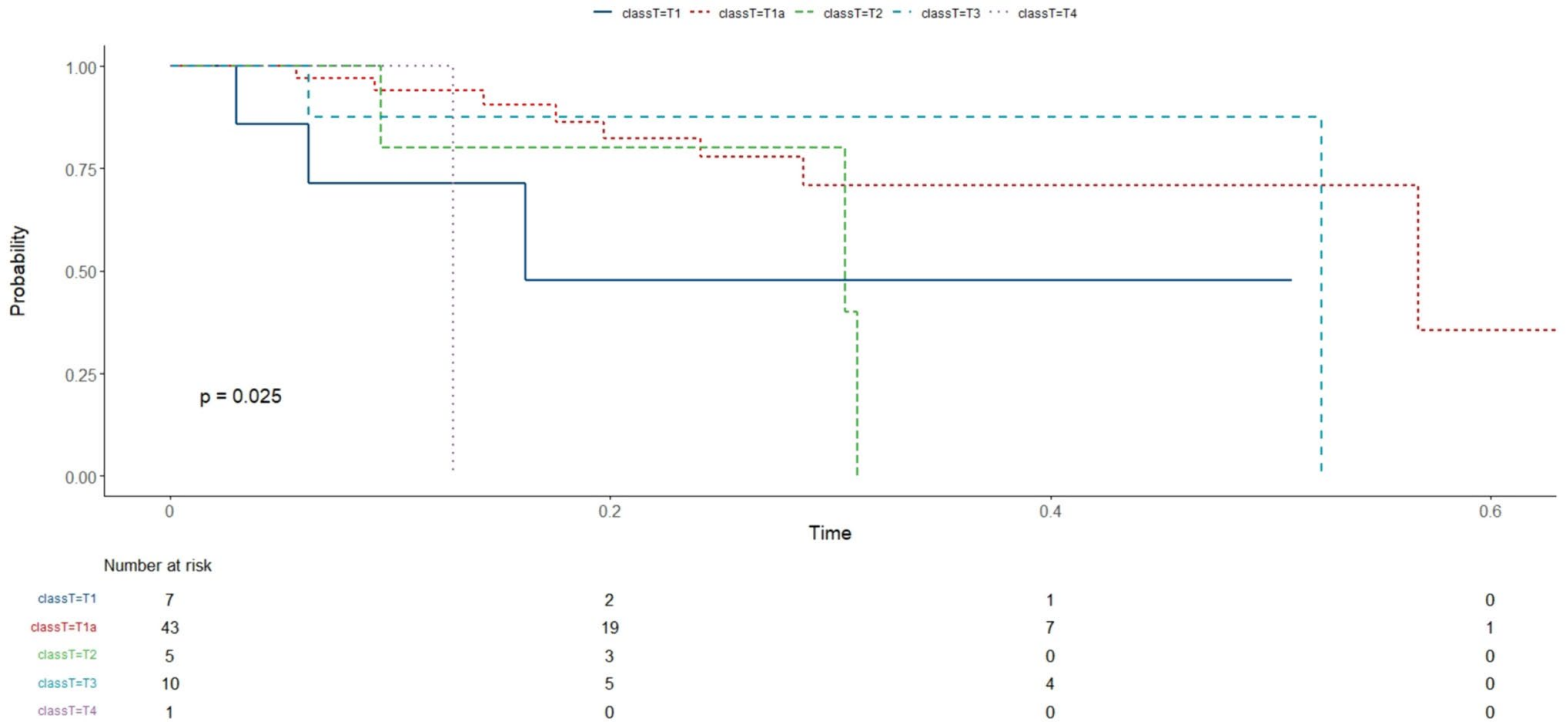
T-classification Kaplan–Meier test. The T-classification of Kaplan–Meier test provides invaluable insights into survival patterns based on the T parameter. This test stratifies patients into different groups according to the size of their tumour and examines their survival probabilities over time

**Fig. 4:**
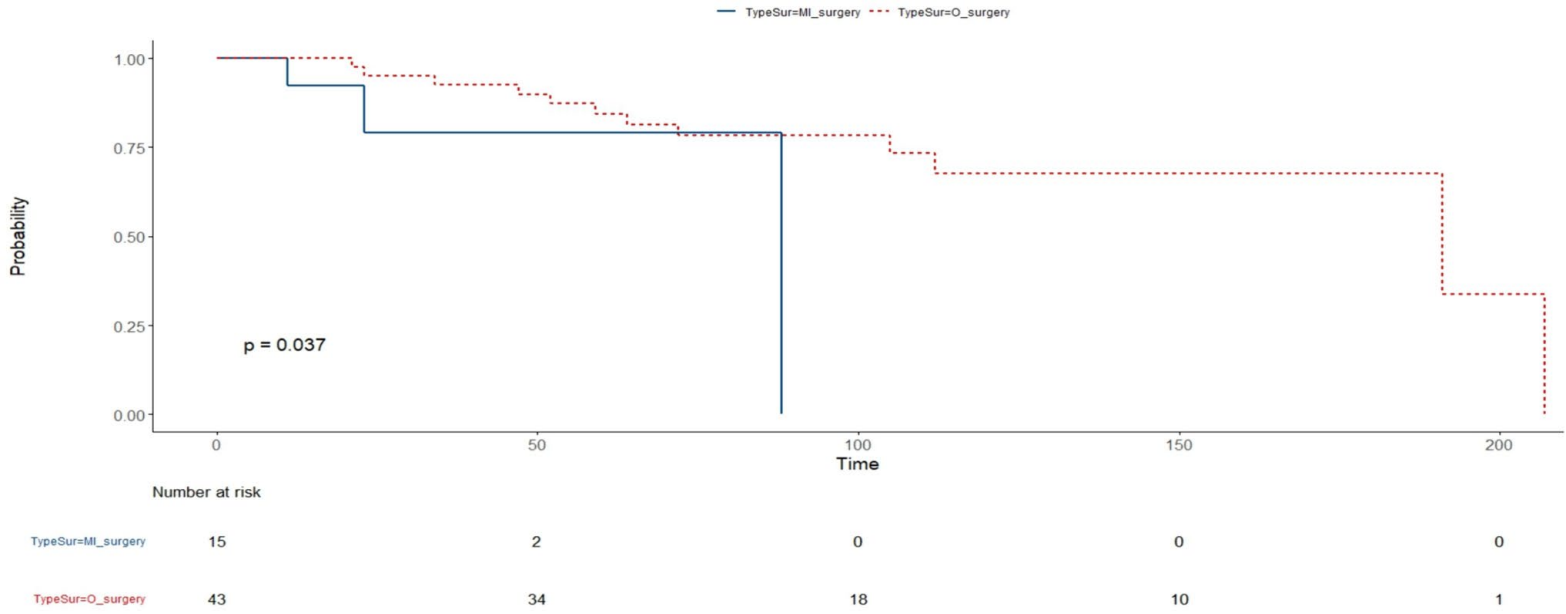
Kind of surgical access, Kaplan–Meier test based on the kind of surgical access provides crucial insights into survival outcomes following different surgical approaches. This test categorizes patients based on the type of surgical access they received and evaluates their survival probabilities over time

Correlation matrix has been considered showing with a gradient of colours and with size of shapes, different levels of correlations between each feature with our outcome (MGFA-PIS) and among them. Therefore, the bigger the square, the stronger is the correlation. Blue indicates positive and red negative correlation. (Fig. 1) One of the approaches to understand our data has been a classification method with random forest. According to the algorithm, we obtained an accuracy of ~ 60%, with 60–70% of sensitivity and 30–40% of specificity. We also analysed the features of patients without neurological response and explored variables predicting a non-neurological response, but the small number of patients did not allow any conclusions (data analysis not shown). Finally, overall survival analysis showed a statistical significance for Kaplan–Meier log-rank test for the presence of capsule microscopic infiltration (Fig. 2), for the TNM staging system (Fig. 3) and for the kind of surgical approach (minimally invasive vs open approach) (Fig. 4), in favor of the open access. No correlation with the neurological outcome (p value 0.51) was found.

We also analysed the features of patients without neurological response. There were seven patients, 4 female (57.1%) and 3 male (42.9%) with a mean age of 67.6 (± 2.5) years. Only one patient had a pericardial infiltration. Mean tumour dimension was 4.7 cm (± 1.2). Five (71.4%) patients underwent RT advjuvant therapy, and the two with worsening had preoperative Osserman score 2a and 2b. Out the five unchanged patients, one had Osserman score 3, one had 4, two had Osserman score 2a, and 1 had 2b.

We explored the possible variables predicting a not neurological response, but the small number of patients did not allow any conclusions.

## Discussion

Thymectomy is a therapeutic option in the management of treatment of patients with myasthenia gravis and positive anti-AchR Ab. However, its effectiveness has only been proven in a randomized controlled trial adopting sternotomic thymectomy among specific inclusion criteria: onset of MG 3 years before surgery, age of 18–65 years, a serum acetylcholine-receptor–antibody level of more than 1.00 nmol/l, and an MGFA score from II to IV [3]. Nevertheless, a substantial proportion of patients in real-world cohorts do not reach complete stable remission after thymectomy indicating that the underlying autoimmune process is sustained in the peripheral lymphatic organs [5–7]. With the significant changes in surgical practice and the increase of elderly population in good clinical condition, the inclusion of MG patients over 65 years to radical thymectomy is questionable and deserves more evidence-based studied. Data in literature already report good results for people aged less than 65, with both open and RATS approach, with some studies reporting a rate of CSR variable between 50 and 60% after surgery, in non-thymomatous patients affected by MG, but evidences are limited and still inconsistent [8–11].

In the present manuscript, we aim to add further data on the neurological efficacy of thymectomy in patients with MG older than 65, who presented with thymic epithelial tumour and for which surgery was indicated for oncological purpose mainly, with few cases in which it was indicated firstly for the neurological outcome (i.e. Osserman score preop 3 or 4).

Firstly, we observed that only a small percentage (10.5%) of patients was neurologically unchanged or had worsening after surgery, according to the MGFA-PIS. The greatest number presented with a minimal manifestation outcome after surgery (65.2%), with a reduction of the need of pharmacological treatment. An encouraging (but still to be consolidated) result also emerged, with a total of 15 patients (22.5%) having complete stable remission (#4) and pharmacological remission (#11).

To the best of our knowledge, not many studies in literature have focused on this particular population of thymoma patients aged over 65, affected with MG and treated with surgery. Romano and colleagues, in a recent study, analysed the neurological outcome after robotic thymectomy for thymoma in patients affected with MG [12]. They obtained improvement of the clinical conditions in 26 patients (76.5%) following the operation: complete stable remission was observed in 5 patients (14.7%), pharmacological remission in 10 (29.4%), and minimal manifestation in 11 (32.3%) [12]. Interestingly, their patients were similar to ours according to the neurological and oncological disease, but the series of Romano and colleagues was composed of patients with a mean age of 52.6 years. The results on neurological outcome are in line with ours, suggesting that surgery is still a good option for patients with these features, but they are still analysing patients under 65 years. Another interesting finding was from Jiao and coworkers, who studied the surgical safety in myasthenia gravis (MG) patients aged > 65 years in a cohort of 564 patients with MG who underwent surgery [13]. In this case, rather than the neurological outcome, they considered the surgical outcome, and concluded that surgical indications should be considered in each elderly MG patient on an individual base. Moreover, most elderly MG patients safely survive the perioperative period, suggesting a low morbidity/mortality rate correlated to MG exacerbation.

On the other hand, Otsuka and colleagues evaluated the long-term clinical outcomes after extended thymectomy in 30 MG patients with or without thymoma [14]. In their cohort, nine patients were over 65 years old. Overall, the symptoms of MG improved in four of the nine (44%) elderly patients only. None of the elderly patients achieved complete stable remission. They concluded that thymectomy can be an option even in elderly patients and suggested performing surgery in a short interval after the onset of MG, better if shorter than 1 year. The population studied by Otsuka and coworkers was very small, and even smaller was the number of patients over 65, so the results are far from being conclusive. In this framework, the present analysis, despite being limited in sample size, is one the biggest reported till now on this topic.

Cunha and coworkers analysed the effect of thymectomy both in patients affected and not affected by thymoma. Their results showed that the cohort affected with thymoma was older than the one of not affected patients (mostly older than 54 years), and they had a good neurological outcome, with a reduction in medication need. In particular, according to their findings, the better outcome was for the ones with an interval between the MG development and surgery shorter than 1 year [15].

Another interesting observation is from Tian and colleagues. In their paper, they analysed the impact of thymoma on neurological outcome in patients affected by thymoma and myastenia gravis. Surprisingly, they found out that thymectomy was associated with a better neurological outcome above all in patients older than 50 years. It is interesting to notice that according to this experience, surgery is associated with a better outcome if MG history before surgery is longer than 1 year, which is in contrast with the findings from Otsuka and Cunha [16].

This is an interesting observation; in particular, in our cohort the mean interval between the onset of MG and surgery was 1.7 years. According to our analysis, no statistical difference was found between patients operated within 12 months from the onset of MG and patients operated after 12 months.

We also tried to evaluate the possible features predicting the neurological outcome, but no statistical association was found when exploring all the clinical pre/postoperative variables and oncological characteristics. In particular, we found no association with the kind of surgical approach (minimally invasive vs open approach), the tumour dimension, the age of onset of the MG, the preoperative pharmacological treatment, and the preoperative Osserman score. These results may suggest that the neurological effect is comparable when performing radical thymectomy by open techniques (mostly sternotomy) or mini-invasive techniques (RATS and VATS) surgery. In this sense, mini-invasive surgery that has been reported to be linked to a better perioperative outcome [9] may be proposed as part of the treatment of MG and can be offered also to patients aged over 65, without a real selection based on other variables (a part from a general clinical selection before surgery). Indeed, if few years ago the surgical approach consisted mainly of standard open approach (sternotomy or thoracotomy), nowadays, the indications for robotic as well as thoracoscopic-assisted thymectomy are being studied [12]. Salahoru and coworkers, in their review, already concluded that minimally invasive approaches as videothoracoscopic surgery or robotic surgery led to a decrease in the length of hospital stay for these patients, with all the advantages related to a shorter stay in the hospital [17]. In particular, robotic thymectomy should improve cosmetic results, reduce postoperative pain, and allow accelerated recovery for patients with MG [18].

A really recent paper, from Nawojowska and colleagues, describes the videothoracoscopic approach in the treatment of myasthenia gravis to be non-inferior compared to the open approach,h offering all the additional benefits of less invasive surgery [19].

Furthermore, in the coming years, the average age of the population is growing, so patients aged over 65 will increase (increasing the number of patients potentially affected by MG in this age).

From a clinical prospective, there is a lack of evidence in this filed and, presently, the opportunity to perform a thymectomy after the clinical onset of MG in patients aged more than 65 years is very questionable and deserves more data for an evidence-based approach. Potentially, nowadays a radical thymectomy, performed by a mini-invasive approach (associated with a faster recovery with less complications compared to the open approach), could be an option also in patients aged over 65 affected with MG, if further analysis will suggest a neurological benefit as well as in the other classes of age. In support of this consideration, Bertoglio and colleagues reported that minimally invasive thymic surgery (both VATS and RATS), even if extended to more than one surrounding organ, is related to a lower incidence of postoperative complications and shorter postoperative length of stay, even in patients that require extended resections [20].

In our series, a superior age limit had not been posed as potentially, if the performance status is adequate, with all the preoperative evaluation confirming that surgery can be offered. Even if, as stated at the beginning, part of the population does not reach a CSR, no features, from our experience, seem to predict the neurological response. Probably, immunological factors, such as the anti-Ach Ab titre, are responsible for the different neurological responses [21, 22]. More oriented studies on the immunological response are necessary to define if a substratification of patients as candidates to surgery can be done.

The main limitation of our study is that our cohort was composed of patients candidate to surgery for oncological purpose and not for neurological reasons. Thus, the neurological outcome after surgery cannot be compared to the one of patients affected only with MG without thymoma, because the biology of the neurological disease could be potentially differentOur results seem to be an interesting starting point to analyse over 65 years old patients affected only with MG and so to extend the indication for surgery in elderly patients. Furthermore, another limitation is the small number of patients, the retrospective nature of our study, and the possible heterogeneity of our data due to the bicentric origin of our sample.

## Conclusion

For patients over 65 affected with MG and thymoma, thymectomy seems to be an effective treatment also to improve neurological symptoms. In our cohort of 66 patients, a neurological response (MM + CSR + PR) was observed in 89.2% of patients, which is a quite encouraging result with only 7 patients presenting with unchanged or worsened response after surgery. At univariate analysis, we found no association with the other analysed features.

These observations may represent the *proof of principle* for exploring this field and proposing to include the use of thymectomy for clinically fit patients aged more than 65 years affected by MG. Our results need to be validated in a larger study composed of patients aged over 65 affected only with MG.

## Data Availability

Data are available on request due to restrictions, e.g., privacy or ethical. The data presented in this study are available on request from the corresponding author.
