# Correction: Thymoma resection and myasthenia gravis: what is the neurological outcome in patients older than 65 years?

**DOI:** 10.1007/s13304-024-01954-9

**Published:** 2024-08-09

**Authors:** Filippo Lococo, Carolina Sassorossi, Giulio Maurizi, Gloria Santoro, Raffaele Iorio, Silvia Falso, Elisa Meacci, Antonio Giulio Napolitano, Maria Teresa Congedo, Giacomo Cusumano, Beatrice Trabalza Marinucci, Giacomo Argento, Marco Chiappetta, Erino Angelo Rendina, Stefano Margaritora

**Affiliations:** 1 Thoracic Surgery Unit, Università Cattolica del Sacro Cuore, Largo F.Vito 1, Rome, Italy; 2 Thoracic Surgery Unit, Fondazione Policlinico Universitario A. Gemelli IRCCS, Rome, Italy; 3 Department of Medical-Surgical Science and Translational Medicine, Sapienza University of Rome, Rome, Italy; 4 Division of Thoracic Surgery, Sant’Andrea University Hospital, Rome, Italy; 5 UOC di Chirurgia Generale, Fondazione Policlinico Universitario A. Gemelli IRCCS, 00168 Rome, Italy; 6 Neurology Unit, Fondazione Policlinico Universitario A. Gemelli IRCCS, Rome, Italy; 7 Department of Neuroscience, Università Cattolica del Sacro Cuore, Rome, Italy; 8 Thoracic Surgery Unit, Policlinico-San Marco Hospital, University of Catania, Catania, Italy

**Correction: Updates in Surgery** 10.1007/s13304-024-01937-w

In this article the author name RAFFAELE IORIO and STEFANO MARGARITORA was incorrectly written as RAFFELE IORIO and MARGARITORA STEFANO.

The original article has been corrected.

